# Sarcoglycans Role in Actin Cytoskeleton Dynamics and Cell Adhesion of Human Articular Chondrocytes: New Insights from siRNA-Mediated Gene Silencing

**DOI:** 10.3390/ijms26125732

**Published:** 2025-06-15

**Authors:** Antonio Centofanti, Michele Runci Anastasi, Fabiana Nicita, Davide Labellarte, Michele Scuruchi, Alice Pantano, Josè Freni, Angelo Favaloro, Giovanna Vermiglio

**Affiliations:** 1 Department of Biomedical, Dental Sciences and Morphofunctional Imaging, University of Messina, 98125 Messina, Italy; antonio.centofanti@unime.it (A.C.); davidelabellarte99@gmail.com (D.L.); angelo.favaloro@unime.it (A.F.); gvermiglio1@unime.it (G.V.); 2 Department of Maxillo-Facial Surgery, University of Sapienza, 00161 Rome, Italy; michele.runci@gmail.com; 3 Department of Clinical and Experimental Medicine, University of Messina, 98125 Messina, Italy; mscuruchi@unime.it (M.S.); alice.pantano@unime.it (A.P.)

**Keywords:** sarcoglycan, focal adhesions, chondrocyte, immunofluorescence

## Abstract

Chondrocytes maintain cartilage integrity through coordinated regulation of extracellular matrix (ECM) synthesis and remodeling. These processes depend on ECM dynamic interactions, mediated by integrin-based focal adhesions and associated cytoskeletal components. While the roles of core adhesion proteins are well described, the involvement of sarcoglycans (SGs) remains unclear in chondrocytes. Drawing parallels from striated muscle, where the SG subcomplex stabilizes the sarcolemma, we hypothesized that SGs similarly integrate into chondrocyte adhesion complexes. This study investigated the SGs (α, β, γ, δ) expression with cytoskeletal and adhesion proteins, including actin and vinculin, in human chondrocytes cultured by immunofluorescence, qPCR, and siRNA-mediated silencing. All four SG isoforms were expressed in the cytoplasmic and membrane domains, with enrichment at focal adhesion sites. Double labeling revealed SG colocalization with F-actin stress fibers and vinculin, indicating integration into the core adhesion complex. Silencing of each SG resulted in disrupted actin stress fibers, diffuse vinculin distribution, reduced focal plaque number, and a change in cell morphology. These findings support the hypothesis that SGs regulate actin cytoskeletal dynamics and focal contact stabilization. Loss of SG function compromises chondrocyte shape and adhesion, highlighting the importance of these glycoproteins also in non-muscle cells.

## 1. Introduction

Chondrocytes are specialized cells responsible for maintaining cartilage homeostasis by regulating the synthesis, remodeling, and degradation of the extracellular matrix (ECM) [1,2,3]. These functions rely heavily on dynamic interactions between the cell and its surrounding matrix, which are mediated by complex molecular structures that anchor the cytoskeleton to the cell membrane and facilitate communication with the ECM [4,5,6].

A central feature of chondrocyte–ECM interaction is the formation of focal adhesions, complex protein assemblies that orchestrate both structural and signaling functions [7,8]. Integrins are key receptors in this process, linking ECM ligands to the intracellular cytoskeleton [8,9,10,11]. This process begins with the clustering of integrins at sites of ECM contact, which initiates a cascade of intracellular signaling events. A crucial aspect of this cascade is the reorganization of the actin cytoskeleton, where filaments assemble into stress fibers that connect to focal adhesion sites, enabling the cell to generate contractile forces and adapt its morphology in response to mechanical stimuli [12,13]. Upon ligand binding, integrins recruit adaptor proteins such as talin and vinculin, which mediate the formation and stabilization of focal adhesion sites [14,15]. Talin binds to the cytoplasmic tail of β-integrins, inducing conformational changes that activate the receptor and promote further recruitment of additional adhesion proteins [16,17]. Vinculin is subsequently recruited to reinforce the linkage between talin and filamentous actin, contributing to the mechanical stability and maturation of adhesion sites [18].

In addition to the well-established components of focal adhesions, other membrane-associated complexes may also contribute to cytoskeletal organization and adhesion. Among these candidates, sarcoglycans (SGs), typically studied in the context of muscle cells, are transmembrane proteins that form a subcomplex of the dystrophin–glycoprotein complex [19], linking the ECM to the cytoskeleton via actin-binding elements to maintain cellular structure and membrane integrity [20,21]. Although extensively studied in the context of muscular dystrophies, the non-muscle specificity of SGs has been recently reported in several studies [22,23]. This broader expression pattern suggests that SGs may play various roles, including contributing to the structural organization of adhesion complexes and regulating cell–matrix interactions in different cell types.

In vitro studies commonly rely on human primary chondrocytes, which are often cultured in a monolayer to facilitate molecular and cellular analyses. However, under these conditions, chondrocytes undergo a well-documented process of dedifferentiation [24,25]. This transition is characterized by a shift in gene expression from cartilage-specific markers, such as collagen type II and aggrecan, to fibroblast-like markers, including collagen type I, accompanied by morphological changes and the development of prominent actin stress fibers [26,27]. This dedifferentiated phenotype provides a simplified model for investigating intracellular protein interactions and cytoskeletal dynamics. Therefore, in this study, we aimed to examine the expression of the α-, β-, γ-, and δ-SG isoforms in human chondrocytes cultured from articular cartilage. Through co-immunolabeling with actin and vinculin, we examined the potential integration of SGs into adhesion complexes and in cytoskeletal dynamics. In addition, we used siRNA-mediated silencing to explore the effects of SG depletion on actin organization and focal adhesion structure.

## 2. Results

### 2.1. Immunofluorescence and RT-PCR on Control Chondrocytes

**Figure 1:**
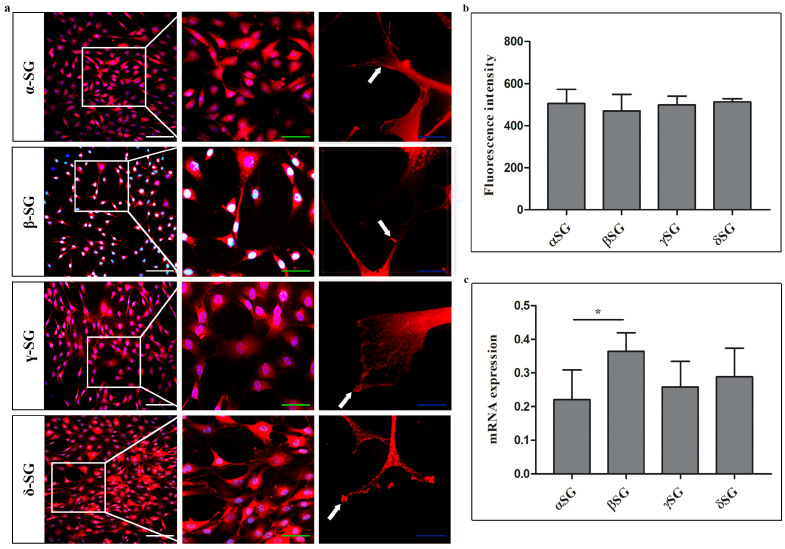
(**a**) Compound panel of single immunofluorescence reactions using anti-α-, β-, γ-, and δ-SGs (red channel). Magnifications: 10×, 20×, and 60× (crop). At 60× magnification, all isoforms revealed a fluorescence pattern at the extremities of plasma membrane elongation, where they interact with the substrate (white arrows). Scale bars: 100 μm (white), 50 μm (green), 25 μm blue. (**b**) Semi-quantitative evaluation of fluorescence intensity for each SG isoform. (**c**) mRNA expression for each SG by qRT-PCR. Numerical data are expressed as mean and standard deviation (SD). *p*-values: * *p* < 0.05.

Immunofluorescence results of single localization reactions showed that all tested SG isoforms (α, β, γ, and δ) are expressed in control cells (Figure 1a). The fluorescence pattern was located at both the cytoplasmic and plasma membrane levels. At 60× magnification, all isoforms revealed a fluorescence pattern at the extremities of plasma membrane elongation, where they interact with the substrate (white arrows). No significant differences in fluorescence intensity values for each isoform were observed (Figure 1b). The qPCR results from control chondrocytes showed detectable mRNA levels for the isoforms assessed, revealing significant differences between β-SG and α-SG, as well as between β-SG and γ-SG (Figure 1c).

**Figure 2:**
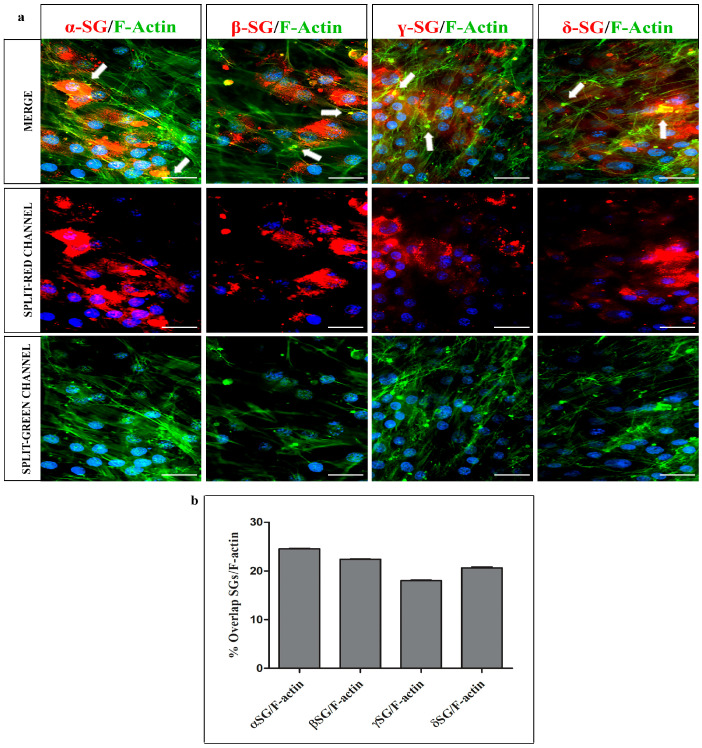
(**a**) Compound panel of double immunofluorescence reactions using antibodies anti-α-, β-, γ-, and δ-SGs (red channel) and F-actin (green channel) and nuclei stained with DAPI (blue channel). It is possible to observe the colocalization between SGs and actin mainly at the plasma membrane elongation of the cells (white arrows). (**b**) Graphic showing the overlap percentage between SGs and F-actin, obtained by ImageJ analysis. Magnifications: 20×. Scale bar: 50 μm.

The double localization reactions of F-actin and SGs revealed a well-organized actin cytoskeleton, characterized by distinct stress fibers (Figure 2). Colocalization among all tested SGs and F-actin was particularly evident at the extremities of plasma membrane elongation, as demonstrated by the yellow fluorescence representing the overlap of both green and red channels (white arrows). The colocalization was assessed by ImageJ, showing the following overlap percentage between each SG and F-actin: 24.54%, 22.4%, 18.01%, and 20.6% for α-, β-, γ-, and δ- SGs, respectively.

**Figure 3:**
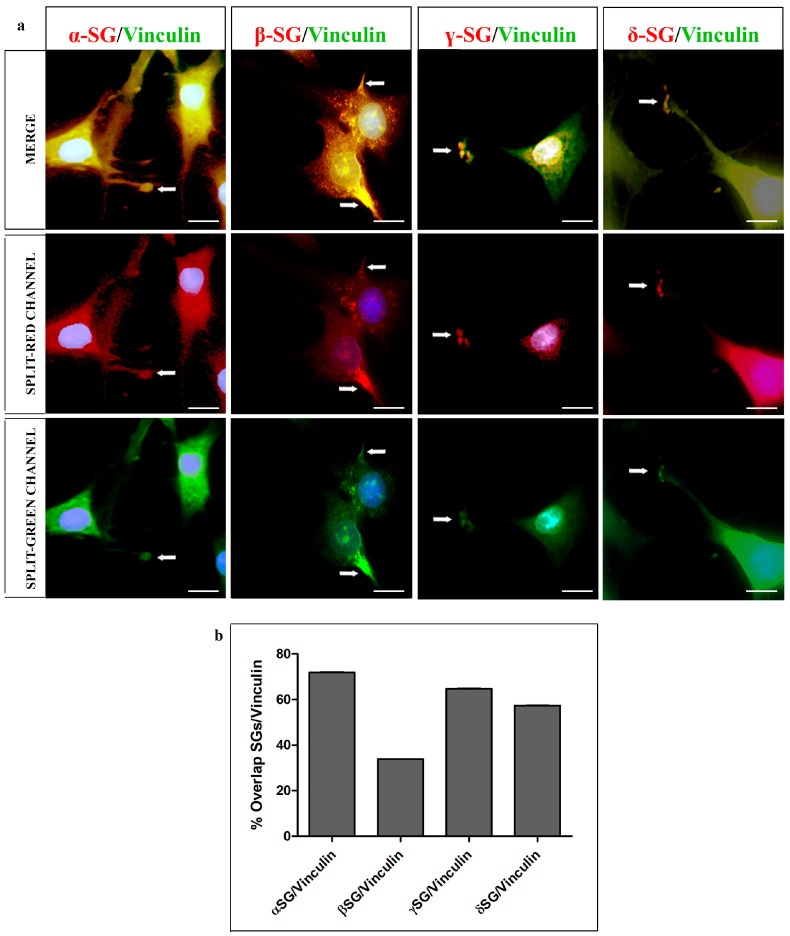
(**a**) Compound panel of double immunofluorescence reactions using antibodies anti-α-, β-, γ-, and δ-SGs (red channel) and vinculin (green channel) and nuclei stained with DAPI (blue channel). It is possible to observe the colocalization between SGs and vinculin (yellow fluorescence), both at the plasma membrane and at the focal plaque level (white arrows). (**b**) Graphic showing the overlap percentage between SGs and vinculin, obtained by ImageJ analysis. Magnifications: 60×. Scale bar: 20 μm.

The double immunoreactions for SG isoforms and vinculin showed that these proteins colocalized at both the cell body and the plasmalemma elongations, corresponding to focal adhesion sites (Figure 3, white arrows). This colocalization was observable through yellow fluorescence. The colocalization was assessed by ImageJ, showing the following overlap percentage between each SG and vinculin: 71.95%, 33.84%, 64.71%, and 57.37% for α-, β-, γ-, and δ- SGs, respectively.

### 2.2. Immunofluorescence on Control and siRNA-Transfected Cells

**Figure 4:**
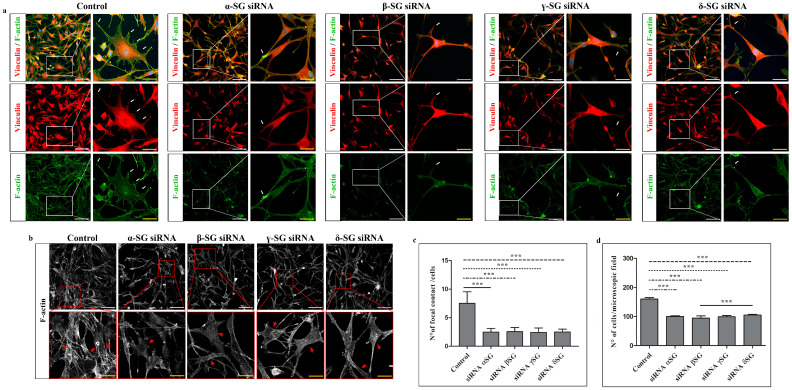
(**a**) Compound panel of double immunofluorescence reactions using antibodies against vinculin (red channel) and F-actin (green channel) in control chondrocytes and in those treated with α-, β-, γ-, δ-SGs siRNA. Magnifications: 20× and 60×. Scale bars: 50 μm (white scale), 20 μm (yellow scale). (**b**) Compound panel of F-actin pictures in grayscale obtained by ImageJ to better show the stress fibers (red arrows) both in control and siRNA groups. (**c**) Semi-quantitative analysis of cell number for the microscopic field and (**d**) focal contacts per cell, both in control and SG-silenced chondrocytes. Numerical data are expressed as mean and standard deviation (SD). *p*-values: *** *p* < 0.001.

Immunofluorescence microscopy revealed markedly altered morphology in chondrocytes after 24 h of siRNA-mediated knockdown of α-, β-, γ-, and δ-SGs (Figure 4a). Control cells exhibited a characteristic polygonal shape, and well-detectable F-actin stress fibers oriented in all directions (Figure 4b). The vinculin expression pattern was localized mainly at cell–substrate interactions, colocalizing with F-actin in areas corresponding to well-defined focal adhesion plaques (Figure 4a). In contrast, SG-silenced chondrocytes exhibited a thin, triangular, elongated morphology with F-actin stress fibers hardly detectable if compared to the control (Figure 4a,b). In addition, the number of visible focal adhesion contacts declined sharply (Figure 4a). Moreover, a statistically significant reduction in a number of cells/microscopic field was observed when compared to the control, as shown by the graphic (Figure 4c); the reduction in a number of cells for the microscopic field is stronger in α-, β-, and γ-SGs silencing when compared to the δ-SG one.

After a count of the focal plaques for both control and SG-silenced chondrocytes, we obtained evidence of a strong decrease in focal plaques in silenced chondrocytes (Figure 4d). In detail, it is possible to observe in the control chondrocytes a mean of 7,52 focal plaques per cell; the following means of focal plaque numbers for cells were found for each silenced SG: 2,48 (α-SG siRNA), 2,54 (β-SG siRNA), 2,43 (γ-SG siRNA), 2,5 (δ-SG siRNA).

**Figure 5:**
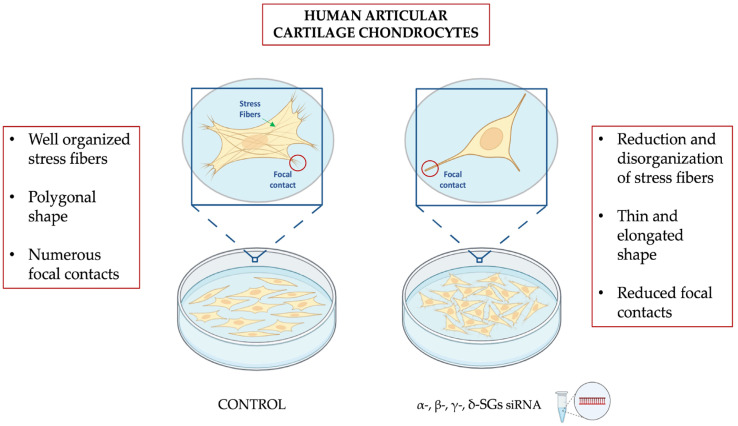
Representation of our data showing the impact of SGs silencing in human articular cartilage chondrocytes. This picture was obtained using BioRender (https://www.biorender.com/ accessed on 10 April 2025).

Figure 5 illustrates our data on the effects of SG silencing in human articular cartilage chondrocytes.

## 3. Discussion

Chondrocytes from articular cartilage are highly specialized cells that exist in a mechanically dynamic environment, where their ability to perceive and respond to external forces is essential for maintaining cartilage integrity [28,29,30]. While many structural and signaling proteins involved in cell–ECM and cell–substrate interactions have been characterized, the potential role of SGs, traditionally associated with muscle tissue, has remained unexplored in cartilage. Our study demonstrates for the first time that four SG isoforms (α, β, γ, δ) are expressed in human articular cartilage chondrocytes. In detail, SGs were identified at the mRNA level using quantitative PCR, supporting the endogenous expression of these proteins. They were also demonstrated at the protein level by immunofluorescence, with a characteristic localization pattern at both the cytoplasmic and membrane levels. Although differences between isoforms were detected at mRNA levels, the fluorescence intensity is similar across isoforms, which supports a coordinated role, rather than a function restricted to specific SG types.

The double localization reactions with F-actin demonstrated a clear colocalization of SGs with a well-organized actin cytoskeleton, particularly at the cell’s periphery and in regions where membrane elongation occurs. Interestingly, it is also the colocalization of SGs with vinculin, further supporting the involvement of SGs in focal adhesion complexes. These observations draw a compelling parallel to costameres in skeletal and cardiac muscle cells, which are complexes that stabilize the cytoskeleton by interacting with integrins and other structural proteins, thereby ensuring the transmission of mechanical force during muscle contraction.

The most revealing are the results from the transfection experiments, where silencing of all SG isoforms led to a clear change in cell shape and cell number for the microscopic field, as well as a reduction in focal contacts per cell. These alterations could result from altered F-actin and vinculin organization. F-actin plays a crucial role in forming a network beneath the plasma membrane, providing mechanical support and tension; that, in turn, helps to maintain a polygonal shape by balancing forces between neighboring cells and the underlying substrate [31,32]. Moreover, F-actin also forms stress fibers that link to focal adhesion complexes, like vinculin, which in turn connect to integrins anchored in the ECM; these sites are crucial for the stable attachment of the cell to the matrix [33]. Vinculin is a mechanosensitive protein that, in its resting state, is autoinhibited [34,35]; when F-actin forms stress fibers and generates tension, this force unfolds talin, exposing vinculin-binding sites and recruiting vinculin to focal adhesion. Based on our results, it is possible to suggest that SGs in cultured chondrocytes could play a key role in actin cytoskeleton dynamics and cell adhesion.

Our data also show that in the absence of SGs, there are fewer cells in the microscopic field compared to the control. Cell proliferation is regulated not only by biochemical signals but also by mechanical cues. Two key players in this mechano-regulation are F-actin and vinculin. Actin-generated tension influences key signaling pathways such as YAP/TAZ, which promote proliferation at high actin tension [32,36]. When F-actin is well organized and under stress, the cell senses a favorable mechanical environment and is more likely to enter the cell cycle. Also, vinculin mediates activation of pro-proliferative signaling, and when focal adhesions are strong and actin tension is high, proliferation is supported [14]. In SG silencing, the disorganized stress fibers and the compromised focal adhesions could impair the chondrocytes’ ability to sense and respond to mechanical stimuli, leading to reduced proliferation and/or cell death. Although the observations are intriguing, the present study did not assay cell proliferation. Therefore, the reason for the decreased number of cells will remain to be studied.

Overall, our data supports several interrelated roles for SGs in in vitro chondrocytes. As SGs are transmembrane proteins that mediate cell–ECM interactions in muscle [37,38], they could play a pivotal role in chondrocytes as key regulators of actin cytoskeleton dynamics, influencing biological processes such as cell shape and adhesion. Disruption of SG expression appears to adversely affect these processes, which may have implications for tissue homeostasis in vivo.

Although this study provides new data on SGs in human chondrocytes, several limitations should be acknowledged. First, we did not assess whether individual SG isoforms exhibit distinct molecular functions or engage with specific subsets of binding partners within focal adhesions. While our data suggests that SGs could participate in cytoskeletal organization alongside integrins, talin, and vinculin, we did not perform co-immunoprecipitation or other biochemical assays to confirm direct physical interactions. Future research should also incorporate super-resolution imaging to elucidate the nanoscale arrangement of adhesions. Additionally, mechanical stimulation was not applied in this study. As a result, the question of how SG function responds to mechanical cues or contributes to chondrocyte adaptation under load remains open. The current observations are, thus, limited to static conditions and may not fully reflect SG behavior in a physiological or mechanically dynamic environment. Biophysical measurements of adhesion strength and signaling assays following controlled mechanical stimulation could help clarify how SGs influence chondrocyte mechanobiology. Our study also did not include functional assays related to proliferation. Given previous suggestions that SGs are involved in cell survival and tissue integrity, future studies should evaluate whether SGs influence the regulation of proliferation. Moreover, all experiments were conducted in monolayer culture, using dedifferentiated human chondrocytes. While this model offers experimental tractability, it does not fully recapitulate the molecular and structural phenotype of mature chondrocytes in vivo. Future investigations should, therefore, include 3D culture systems, biomimetic scaffolds, or freshly isolated chondrocytes to validate and extend these findings. While our study provides a foundation for understanding SG involvement in cytoskeletal and adhesion-related structures in dedifferentiated chondrocytes, a more integrative approach will be essential to fully understand their biological significance and therapeutic potential in cartilage repair and degeneration.

## 4. Materials and Methods

### 4.1. Cell Culture

Human primary chondrocytes from articular cartilage were obtained from SCIENCELLTM (Carlsbad, CA, USA). Cells were cultured in 75 cm^2^ plastic flasks containing 15 mL of the specific chondrocyte medium to which were added 2,5% FBS, L-glutamine (2.0 mM), and penicillin/streptomycin (100 U/mL), obtained from GIBCO (BRL Life Technologies, Grand Island, NY, USA). Cells were incubated at 37 °C in humidified air with 5% CO2 [39]. Experiments were performed using chondrocyte cultures between the third and fifth passages, with cells seeded in six-well culture plates at a density of 2.5 × 10^5^ cells/well.

### 4.2. Chondrocyte Transfection

Chondrocytes were seeded in an eight-well glass slide system (Nunc™ Lab-Tek™ Chamber Slide System, Thermo Fisher Scientific, Waltham, MA, USA) at a 1.5 × 104 cells/well density. Twenty-four hours after seeding, the culture medium was replaced with OPTIMEM (Life Technologies, Carlsbad, CA, USA). Then, chondrocytes were transfected with SGCA, SGCB, SGCG, and SCGD siRNAs (7 pmol/well) using the EndoFectin™ RNAi transfection kit following the manufacturer’s protocol (GeneCopoeia, Inc., Rockville, MD, USA). A negative control siRNA (NC-siRNA) was also included under the same conditions in the other wells.

### 4.3. RNA Isolation, cDNA Synthesis, and qPCR

Total RNA was isolated from non-silenced chondrocytes using TRIzol Reagent Kit (Thermo Fisher Scientific, Waltham, MA, USA). The first strand of cDNA was synthesized from 5.0 µg of total RNA using the High-Capacity cDNA Reverse Transcription Kit (Thermo Fisher Scientific Waltham, MA, USA), and qPCR evaluation of SGCA, SGCB, SGCG, and SCGD was carried through a real-time PCR system (mod 7500, Applied Biosystems Inc., Carlsbad, CA, USA), using the PowerUp SYBR Green Master Mix (Applied Biosystems Inc., Carlsbad, CA, USA). The amplified PCR products were quantified by measuring the calculated cycle thresholds (Ct) of SGCA, SGCB, SGCG, SCGD, and β-actin mRNA. In addition, a melting curve analysis was always performed to verify the specificity of the reactions. After data normalization, using ß-actin as a housekeeping gene, mRNA expression in samples was calculated using the 2−ΔCT method. The sequences of primers used are reported in Table 1.

### 4.4. Immunofluorescence on Cells

Both control cells and silenced cells were fixed on glass coverslips with Immunofix (paraformaldehyde 4%) (Bio-Optica Milano Spa, Milano, Italy) for 30′ and washed five times with phosphate-buffered saline (PBS). Cells were treated with two pre-incubation steps: first with 0.3% Triton X-100 in PBS at room temperature (r.t.) for 10 min to permeabilize the cell membranes, and second with 1% bovine serum albumin (BSA) at r.t. for 20 min to obstruct non-specific antigen-binding sites.

In control cells, we performed two double immunoreactions: the first one between each SG isoform tested and vinculin antibodies; the second one between each SG and F-actin. The cells were incubated with primary antibodies overnight at 4 °C. The following primary antibodies were used: mouse monoclonal anti -α, -β, -γ, and -δ SG (diluted 1:100 in PBS-Albumin 1% solution; Santa Cruz Biotechnology, Inc., Dallas, TX, USA), rabbit polyclonal anti-vinculin (diluted 1:100 in PBS-Albumin 1% solution; Sigma-Aldrich, Saint Louis, MO, USA), and actin-phalloidin FITC (diluted 1:100 in PBS-Albumin 1% solution; Sigma-Aldrich, Saint Louis, MO, USA) The α-, β-, γ-, and δ-sarcoglycans primary antibodies were detected using Texas Red-conjugated secondary antibody (diluted 1:100 in PBS-Albumin 1% solution; Jackson ImmunoResearch Laboratories, West Grove, PA, USA), and F-actin and vinculin primary antibodies were demonstrated with FITC fluorochrome anti-rabbit (1:100 dilution; Jackson ImmunoResearch Laboratories, West Grove, PA, USA).

In silenced cells, one set of double immunoreactions was performed, and the following primary antibodies were used: rabbit polyclonal anti-vinculin (diluted 1:100 in PBS-Albumin 1% solution; Sigma-Aldrich, Saint Louis, MO, USA) and actin-phalloidin FITC (diluted 1:100 in PBS-Albumin 1% solution; Sigma-Aldrich, Saint Louis, MO, USA). The vinculin primary antibody was demonstrated using Texas Red-conjugated secondary antibody (diluted 1:100 in PBS-Albumin 1% solution; Jackson ImmunoResearch Laboratories, West Grove, PA, USA).

### 4.5. Confocal Laser Microscope Observation

Both cells and human articular cartilage sections were examined, and images were obtained utilizing a Zeiss LSM 510 DUO (Carl Zeiss, Jena, Germany) confocal laser scanning microscope. All photos were digitized at a resolution of 8 bits into an array of 2048 × 2048 pixels. Optical sections of fluorescent specimens were acquired using a helium–neon (HeNe) laser (Carl Zeiss, Jena, Germany) with a wavelength of 543 nm at a scanning speed of 62 s and up to 8 averages. Sections were achieved using a pinhole of 250 μm. Contrast and brightness were determined by analyzing the most luminously labeled pixels and selecting the parameters that facilitated clear visualization of structural features while preserving the maximum pixel intensity (~200). Digital images were cropped, and figure montages were assembled using Adobe Photoshop 12.1.

### 4.6. Image Analysis

Fluorescence image processing and quantification were performed using ImageJ software (version 1.54, NIH, Bethesda, MD, USA) [40]. For each SG isoform, mean fluorescence intensity was measured in 10 randomly selected microscopic fields. The “Measure” function was applied after defining regions of interest (ROIs) corresponding to individual cells or subcellular regions. To assess colocalization between each SG and vinculin or F-actin, the “Split Channels” function was used to separate the fluorescence signals. Each channel was subjected to thresholding using the “Threshold” tool to isolate the specific signal of interest. Colocalization analysis was performed using the “Coloc 2” plugin, and results were expressed as the percentage of overlapping area relative to the total signal.

Focal adhesions were identified as discrete vinculin-positive structures, typically localized at the cell periphery. To quantify focal adhesions, images were first converted to 8-bit grayscale and thresholded to highlight vinculin-positive areas. The “Set Measurements” function was configured to include area and particle count, and the “Analyze Particles” tool was used to count focal adhesion structures automatically. Measurements were performed on 10 randomly selected microscopic fields and normalized per cell.

To better visualize actin stress fibers, the green fluorescence channel corresponding to F-actin was converted to 8-bit grayscale using “Image > Type > 8-bit”, and contrast was adjusted with the “Brightness/Contrast” tool to enhance fiber definition without altering quantitative data.

### 4.7. Statistical Analysis

The normal or non-normal data distribution was assessed using the Shapiro–Wilk test. A one-way ANOVA test was used to evaluate the values of fluorescence intensity and mRNA expression for each SG [41]. The Kruskal–Wallis test compared the number of focal contacts before and after SGs silencing. When a significant difference was detected, the appropriate Turkey HSD and Dunn post hoc tests were used, considering the homogeneity of data variances. Numerical values were expressed as mean and standard deviation (SD). Statistical analyses were performed using SPSS for Windows, version 25.0 (IBM, New York, NY, USA). *p* < 0.05 was accepted as the value of statistical significance [42]. Graphics were created using GraphPad Prism (version 10) and displayed all the statistical results.

## 5. Conclusions

Our data identify SGs as a possible novel regulator of actin cytoskeleton dynamics and focal contacts. Their loss disrupts adhesion integrity and cytoskeletal organization, suggesting a role in the biological processes of chondrocytes. Targeting SG-mediated pathways may hold therapeutic potential for preserving cartilage resilience and delaying structural tissue alterations.

## Figures and Tables

**Table 1:** The sequences of primers utilized.

Gene	Species	Sequence (5′→3′)
ACTB	Human	F: 5′-GCTGTCTCTCTATGCCTCTGGA-3′ R: 5′-CCAGATCCAGACGCATGAT-3′
SGCA	Human	F: 5′-ACTTCCGCGTTGACTGGT-3′ R: 5′-AGTGGGTGGGCAGAAGAA-3′
SGCB	Human	F: 5′-CTGACATGGGAGTGATCCAC-3 R: 5′-TGAGCTTTGTTGTCCCTTGC-3′
SGCG	Human	F: 5′-CTGTAAATGCGCGCAACTC-3′ R: 5′-AAATAGTGGCTTGCCGTCGT-3′
SGCD	Human	F: 5′-ATCAATGCAGAAGCTGGCA-3′ R:5′-GATCCATGAGGCAGTCTAGGT-3′

## Data Availability

All data are included within the article.
